# Exploration of biomarkers for efficacy evaluation of traditional Chinese medicine syndromes of acute exacerbation of chronic obstructive pulmonary disease based on metabolomics

**DOI:** 10.3389/fphar.2024.1302950

**Published:** 2024-01-26

**Authors:** Zhang Hailong, Si Yimei, Du Yan, Liu Xinguang, Li Jiansheng

**Affiliations:** ^1^ Co-construction Collaborative Innovation Center for Chinese Medicine, Respiratory Diseases by Henan and Education Ministry of P. R. China, Henan University of Chinese Medicine, Zhengzhou, China; ^2^ The First Affiliated Hospital of Henan University of Chinese Medicine, Zhengzhou, China; ^3^ Henan Key Laboratory of Chinese Medicine for Respiratory Disease, Henan University of Chinese Medicine, Zhengzhou, China

**Keywords:** biomarkers, AECOPD, efficacy evaluation, TCM syndrome, metabolomics

## Abstract

**Background:** Traditional Chinese medicine (TCM) is effective for the treatment of acute exacerbation of chronic obstructive pulmonary disease (AECOPD); however, there is no objective index for the evaluation of TCM syndrome efficacy. This study aimed to screen biomarkers related to the efficacy of TCM syndrome using metabolomics.

**Methods:** We recruited AECOPD patients with phlegm-heat congesting lung (PH)/phlegm-damp amassing lung (PD) syndrome and treated them with Chinese herbal medicine (Qingre Huatan or Zaoshi Huatan granules) in addition to conventional medicine for 7 days. Data on clinical symptoms and sign scores, modified British Medical Research Council (mMRC), COPD assessment test (CAT), and inflammation indicators, including white blood cell (WBC) count, percentage of neutrophil count (NEU%), and C-reactive protein (CRP), were collected before and after treatment to evaluate the therapeutic effect. Serum samples were collected before and after treatment for metabolomic analysis to screen differential metabolites.

**Results:** A total of 69 patients with AECOPD were enrolled, including 41 and 28 patients in the PH and PD groups, respectively. The clinical symptoms and sign scores, CAT, mMRC, NEU%, and CRP levels after treatment were lower than those before treatment in both groups (*p* < 0.05). Serum metabolomics analysis showed that there were 13 differential metabolites in the PH group and 16 differential metabolites in the PD group before and after treatment (*p* < 0.05, variable importance projection (VIP) ≥ 1.00). In the PH group, lysophosphatidylcholine (LPC) (16:0), LPC (17:1), LPC (18:3), LPC (18:2), and LPC (17:0) negatively correlated with clinical symptoms and sign scores (*p* < 0.05); LPC (16:0), LPC (17:1), LPC (16:1), and LPC (17:0) negatively correlated with WBC (*p* < 0.05) and NEU% (*p* < 0.05); and LPC (16:0) negatively correlated with CRP levels. In the PD group, L-phenylalanine positively correlated with CRP levels (*p* < 0.05), and 2-methylbutyroylcarnitine positively correlated with clinical symptoms and sign (*p* < 0.05) and CAT scores (*p* < 0.05). DL-carnitine positively correlated with clinical symptoms and sign scores (*p* < 0.05).

**Conclusion:** Serum metabolites may be potential indicators to objectively evaluate the efficacy of TCM syndromes; however, further large controlled trials are required to verify these findings.

## 1 Introduction

Chronic obstructive pulmonary disease (COPD) is a worldwide public health challenge due to its high prevalence, disability and mortality rates (GBD, 2015 Chronic Respiratory Disease Collaborators, 2017; GBD, 2015 Disease and Injury Incidence and Prevalence Collaborators, 2016; GOLD, 2023). In China, the overall prevalence of COPD was 13.7% in adults aged 40 years or older in 2015, while the total number of patients was nearly 100 million (Wang et al., 2018). COPD is the third leading cause of death globally and ranked the seventh as a cause of disease burden as measured by disability-adjusted life years (DALYs) in 2019 (GBD Chronic Respiratory Disease Collaborators, 2020). Acute exacerbation of COPD (AECOPD) is a major event affecting the natural course of COPD. Patients with COPD have about 0.5–3.5 acute exacerbations per year, which have a serious negative impact on the quality of life, disease progression, and socioeconomic burden of the patients. According to evidence-based guidelines, early detection and timely treatment of deterioration are key to ensuring the best outcomes and reducing the burden of chronic obstructive pulmonary disease (Expert group on Acute Exacerbations of Chronic Obstructive Pulmonary Disease, 2017; GOLD, 2023).

Studies have shown that Chinese herbal medicines are safe and effective in the treatment of AECOPD, and that the treatment of AECOPD by integrating Chinese and Western medicine is superior to conventional Western medicine in terms of increasing clinical efficiency, shortening hospital stay, relieving symptoms, improving lung function, and alleviating inflammation (Meng, 2017; Bai et al., 2022). Therefore, Traditional Chinese medicine (TCM) can be used as a supplementary or alternative treatment for patients with AECOPD (Li et al., 2020a; Ma, 2022; GOLD, 2023). TCM syndromes, as the basis for the diagnosis and treatment of diseases, summarize the etiology, location, disease, and condition of the body at a certain stage in disease development, which is the key index for evaluating the clinical efficacy of TCM. Changes in the multilevel metabolic networks of the body are important (Tao et al., 2018; Tao et al., 2020). The evaluation index of the curative effect of TCM syndromes is the main component of the TCM clinical curative effect evaluation system (Guo et al., 2017). At present, the main criterion for judging the efficacy of TCM syndromes is to evaluate the degree of improvement in TCM syndromes before and after treatment. The TCM syndrome scores and syndrome efficacy evaluation scale are widely used to evaluate the intervention effect of TCM; however, due to many problems, including unclear positioning of the measured outcome level, confusion between diagnosis and evaluation attributes of TCM syndromes, and difficulty in measuring dynamic changes of TCM syndromes, the objectivity and scientificity of TCM syndromes efficacy evaluation has always been controversial (Hu, 2012).

Metabolomics technology can accurately describe the dynamic changes of in endogenous small-molecule metabolites in the body to understand the overall functional state of the body. It has phase, dynamic, and integral characteristics, which are consistent with the TCM syndrome concept (Liu et al., 2022). It has been widely used for disease diagnosis (Wang et al., 2013), differential diagnosis (Deja et al., 2014), and therapeutic interventions (Ren et al., 2016). Several studies have explored biomarkers and pathophysiological mechanisms associated with the diagnosis and classification of TCM syndromes in AECOPD (Yang et al., 2018; Zhang et al., 2020). However, there is a lack of biomarker research on the efficacy of TCM syndromes.

We hypothesized that AECOPD patients with different syndromes have different metabolic profiles before and after treatment, and that metabolomic analysis based on LC-MS can screen biomarkers related to the evaluation of TCM syndrome efficacy. The most common TCM syndromes in patients with AECOPD are phlegm-heat congesting lung (PH) and phlegm-damp amassing lung (PD) (Li et al., 2017). Our previous researches showed that Qingre Huatan granules and Zaoshi Huatan granules have good clinical efficacy in the treatment of PH and PD in AECOPD and can significantly improve clinical symptoms, improve lung function, and reduce inflammatory reactions (Li et al., 2010; Li et al., 2010; Li et al., 2010; Li et al., 2011; Li et al., 2020b). Therefore, this study analyzed the serum metabolomics of AECOPD patients with PH and PD syndrome to identify biomarkers with high specificity and sensitivity and to provide an objective basis for TCM syndrome differentiation and treatment and TCM syndrome efficacy evaluation of AECOPD.

## 2 Materials and methods

### 2.1 Polulation

This study was approved by the Ethics Committee of the First Affiliated Hospital of Henan University of Traditional Chinese Medicine according to the Declaration of Helsinki (2017HL-086-01). All the participants signed an informed consent form before participanting the study.

The subjects were AECOPD patients aged from 40 to 80 years old in the general ward of the respiratory department of the First Affiliated Hospital of Henan University of Chinese Medicine from August 2018 to September 2019. All subjects were diagnosed according to “Global Initiative for Chronic Obstructive Lung Disease (GOLD): global strategy for the diagnosis, management, and prevention of COPD (2017 REPORT)” (GOLD, 2017) and “Expert Consensus on the Diagnosis and Treatment of Acute Exacerbation of Chronic Obstructive Pulmonary Disease (AECOPD) in China (Updated in 2017)” (Expert group on Acute Exacerbations of Chronic Obstructive Pulmonary Disease, 2017), and divided into the PH group and PD group based on TCM syndrome differentiation referred to the “Diagnostic Criteria for TCM Syndromes of Chronic Obstructive Pulmonary Disease (2011 edition)” (Li et al., 2012).

The exclusion criteria were as follows: a) pregnant and lactating women; b) patients with severe cardiac insufficiency; c) patients with bronchial asthma, bronchiectasis, active tuberculosis, or diffuse panbronchiolitis; d) patients with pneumothorax, pleural effusion, or pulmonary embolism; e) patients with definite neoplastic disease; f) patients with severe liver and kidney disease; g) participating in clinical trials of other interventions.

### 2.2 Intervention

All the participants received conventional Western medical treatment, including controlled oxygen therapy, bronchodilators, glucocorticoids, antibiotics and so on. The specific treatment measures were individualized according to “Expert Consensus on the Diagnosis and Treatment of Acute Exacerbation of Chronic Obstructive Pulmonary Disease (AECOPD) in China (Updated in 2017)” (Expert group on Acute Exacerbations of Chronic Obstructive Pulmonary Disease, 2017). The treatment principles are shown in Supplementary Material. They were administered the Chinese herbal Qingre Huatan granules in the PH group and Zaoshi Huatan granules in the PD group. The Chinese herbal medicine granules were supplied by Jiangyin Tian Jiang Pharmaceutical Co., Ltd. Jiangsu, China. Chinese herbal medicine granules were manufactured in strict accordance with the standards of the Chinese Pharmacopoeia (2010). Each granule type was dissolved in warm water and administered orally at a dose of 10 g twice daily for 14 days. The components of the Chinese herbal medicine granules list in Table 1.

**TABLE 1:** The components of Chinese herbal medicine granules.

Chinese name	Latin name	English name	Amount (g)
Qingre huatan granules			
Gua Lou	Trichosanthis fructus	Snakegourd fruit	15
Qing Ban Xia	Pinelliae Rhizoma Praeparatum Cum Alumine	Alum processed pinellia	9
Zhe Bei Mu	Fritillariae Thunbergii Bulbus	Thunberg fritillary bulb	9
Zhi Zi	Gardeniae fructus	Cape jasmine fruit	9
Sang Bai Pi	Mori Cortex	White mulberry root-bark	12
Huang Qin	Scutellariae Radix	Baical skullcap root	9
Ku Xing Ren	Armeniacae Semen Amarum	Bitter Apricot Seed	9
Bai Tou Weng	Pulsatillae Radix	Chinese pulsatilla root	15
Yu Xing Cao	Houttuyniae Herba	Heartleaf houttuynia herb	15
Chi Shao	Paeoniae Radix Rubra	Peony root	12
Xi Yang Shen	Panacis Quinquefolii Radix	American ginseng	6
Mai Dong	Ophiopogonis Radix	Dwarf lilyturf tuber	12
Chen Pi	Citri Reticulatae Pericarpium	Dried tangerine peel	9
Zaoshi Huatan granules			
Fa Ban Xia	Pinelliae Rhizoma Praeparatum	Processed pinellia tuber	9
Hou Pu	Magnoliae Officinalis Cortex	Officinal magnolia bark	9
Xie Bai	Allii Macrostemonis Bulbus	Longstamen onion bulb	12
Fu Ling	Poria	Indian bread	15
Ting Li Zi	Descurainiae Semenlepidii Semen	Pepperweed seed	9
Zi Su Zi	Perillae fructus	Perilla fruit	9
Jie Zi	Sinapis Semen	Mustard seed	9
Chi Shao	Paeoniae radix rubra	Peony root	12
Ren Shen	Ginseng Radix Et Rhizoma	Ginseng	6
Xi Xin	Asari Radix Et Rhizoma	Manchurian wildginger	2
Chen Pi	Citri Reticulatae Pericarpium	Dried tangerine peel	12

### 2.3 Clinical efficacy evaluation index

Clinical symptoms and sign scores, modified British Medical Research Council (mMRC), COPD assessment test (CAT), and inflammatory indicators, including white blood cell (WBC) count, percentage of neutrophil count (NEU%), and C-reactive protein (CRP) levels, were recorded before and after treatment to evaluate the therapeutic effect. The occurrence of adverse events and electrocardiogram (ECG) and laboratory examinations, such as routine tests of blood, urine, and stool and function tests of the liver and kidney, were used as safety indicators observed and recorded before and after treatment of AECOPD.

### 2.4 Statistical analysis

SPSS software (version 23.0, IBM, United States) was used to build the database and perform statistical analyses. Categorical variables were presented as numbers (percentages). The paired *t*-test was used to assess the continuous variables of a normal distribution, which were described by mean (M) ± standard deviation (SD). The continuous variables of non-normal distribution were described as median (interquartile range [IQR]) and analyzed using the Wilcoxon signed-rank test. A *p*-value of less than 0.05 (*p* < 0.05) was deemed statistically significant for results.

### 2.5 Metabolomics analysis

#### 2.5.1 Biochemical and instrumental investigations

Formic acid (FA), acetonitrile (ACN), and ammonium acetate (NH4OAc) were obtained from Thermo Fisher, United States. Tryptophan-d5, valine-d8, carnitine C8:0-d3, carnitine C16:0-d3, cholic acid-d4, and stearic acid-d3 (98% purity) were purchased from Cambridge Isotope Laboratories (Tewksbury, MA, United States). Lysophosphatidylcholine LPC (19:0) was obtained from Avanti Polar Lipids (Alabaster, AL, United States). Pure water was purchased from Wahaha ((Hangzhou, China).

#### 2.5.2 Serum samples preparation

Fasting blood of all subjects was collected in the early morning before and after treatment and allowed to clot naturally at room temperature, which was then centrifuged at 3,000 r/min at 4°C for 15 min to obtain the serum. The samples were kept at −80°C until analysis.

Frozen serum samples were thawed at 4°C. 100 μL serum was transferred, and 400 μL ACN containing seven internal standards was added to remove the proteins. After centrifugation at 13,000 r/min and 4°C for 15 min, two aliquots (200 μL for each) of the supernatant were transferred and then dried in a vacuum centrifuge. The residues were reconstituted with 40 μL 20% ACN before LC-MS analysis.

Quality control (QC) samples were prepared by mixing 10 μL of each serum sample and pretreated as the real samples. At the beginning of the sequence, eight QC samples were run to balance the instrument system and one QC sample was run every six real samples to monitor the stability of the analysis (Luo et al., 2016).

#### 2.5.3 LC-MS analysis

A Dionex Ultimate 3,000 ultra-performance liquid chromatograph (UPLC) coupled to a Q-Exactive Mass Spectrometer (MS) (Thermo Fisher Scientific, Bremen, Germany) via an electrospray ionization source was used for metabolomic analysis. An ACQUITY BEH C18 column (2.1 mm × 100 mm, 1.7 µm) (Waters, Milford, MA, United States) was employed for chromatographic separation. Mobile phases A and B were 0.1% FA in H_2_O and 0.1% FA in ACN in the electrospray ionization-positive (ESI+) mode, and 5 mM ammonium acetate in water and 5 mM ammonium acetate in 95% ACN in the electrospray ionization-negative (ESI-) mode.

Gradient elution for both modes of analysis was performed as follows: maintained at 5% B for 1 min, then linearly increased to 100% B for 24 min, followed by a 4 min wash at 100% B and a 5 min equilibration at 5% B. The column temperature and flow rate were set at 30°C and 0.35 mL/min, respectively. The injection volume was 2 μL.

MS acquisition was conducted in ESI+ and ESI- modes with the spray voltage at 3.5 and 2.8 kV respectively. The capillary temperature, flow rate of the sheath gas and auxiliary gas, and S-lens RF level were set respectively at 350°C, 40 arbitrary units, 10 arbitrary units, and 55 for both modes. Sequence analysis was performed in full MS mode from 70 to 1,000 m/z at the resolution of 70,000. For identification analysis, the full MS-ddMS2 mode was used, and the parameter settings were as follows: resolution of MS and MS2, 70,000 and 17,500; Top N, 5; dynamic exclusion time, 6 s; normalized collision energy, 20, 40, 60; selection of the top 10 ions for secondary scanning; and dynamic elimination time, 10 s.

#### 2.5.4 Data processing and differential metabolites screening

Raw data were processed using Compound Discoverer 2.1 (Thermo Fisher Scientific Co., Ltd., United States) with an untargeted workflow that included retention time alignment, compound detection, grouping, background annotation, formula prediction, and database search. The peak list was exported and re-integrated using TraceFinder (Thermo Fisher Scientific Co., Ltd., United States). The missing values were removed according to the “80% rule,” the rest of which were filled by the sequential k-nearest neighbor algorithm. The peak area of each compound in the real samples was calibrated using an internal standard, which was selected based on the performance of the internal standards [with minimum relative standard deviation (RSD)] in the QC samples.

The Wilcoxon signed-rank test was first conducted to discern differential compounds using SPSS (version 23.0, IBM, United States) with *p* < 0.05 defined as statistically significant. Multivariate statistical analyses, including principal component analysis (PCA) and partial least squares discriminant analysis (PLS-DA) were performed with SIMCA 14.0 (Umetrics AB, Umea, Sweden). Differential metabolites were further screened based on the variable importance projection (VIP) ≥ 1.00. Permutation tests of 200 times were performed to assess the stability of the model and risk of overfitting.

### 2.6 Identification of differential metabolites and analysis of metabolic pathways

For the identification of differential metabolites, candidates from the m/zCloud database in the search results of Compound Discoverer 2.1 were checked manually. Both the deviation of the precursor ions and matching of the MS/MS spectra were considered. For differential metabolites with similarity matching with carnitine or LPC, the MS/MS spectra were checked for the characterized production of 85.0291 or 184.0733 for carnitine or LPC, respectively, and the precursor ions were searched against HMDB (https://www.hmdb.ca) and LipidMaps (https://www.lipidmaps.org) with a mass tolerance of 10 ppm to determine their structures. Finally, some of the compounds were further validated by comparing their retention times, precursors, and fragment ions with those of the standards. Metabolic pathway analyses were performed via the MetaboAnalyst 5.0 (https://www.metaboanalyst.ca).

## 3 Results

### 3.1 Characteristics of study subjects

We recruited 112 patients with AECOPD, who were divided into the PH group (*n* = 60) and the PD group (*n* = 52) according to their TCM syndromes. However, 43 patients (19 in the PH group and 24 in the PD group) did not have blood samples collected after treatment for personal reasons; therefore, 69 patients were finally included in the study. Baseline characteristics of all patients are shown in Table 2.

**TABLE 2:** Baseline characteristics of the study population.

	PH group (*n* = 41)	PD group (*n* = 28)
Age (years)	66.37 ± 8.49	68.82 ± 6.09
Sex, male/female, n (%)	35(85.4)/6(14.6)	27(96.4)/1(3.6)
Height (cm)	169.66 ± 6.68	169.25 ± 5.66
Weight (kg)	66.00 ± 11.35	66.04 ± 14.03
BMI (kg/m^2^)	22.91 ± 3.56	22.99 ± 4.45
Smoking status, n (%)		
Never	18(43.9)	4(14.3)
Former	15(36.6)	20(71.4)
Current	8(19.5)	4(14.3)
Pack-years	48.41 ± 39.85	48.70 ± 29.35
Passive smoker, n (%)	10(24.4)/31(75.6)	8(28.6)/20(71.4)
Course of COPD (months)	94.66 ± 77.30	113.07 ± 121.20
Number of aggravations in the past year	1.10 ± 1.04	1.18 ± 1.09
Degree of illness, n (%)		
moderate	4(9.8)	1(3.6)
severe	37(90.2)	27(96.4)
Combined diseases, n (%)	21(51.2)/20(48.8)	13(46.4)/15(53.6)
Hypertension	8(38.1)	6(46.2)
Diabetes	5(23.8)	2(15.4)
Coronary heart disease	4(19.0)	7(53.8)

### 3.2 Evaluation of clinical efficacy

In the PH group, the clinical symptoms and sign, mMRC, and CAT scores of patients after treatment were significantly lower than those before treatment (*p* < 0.05). The NEU% and CRP levels after treatment were significantly lower than those before treatment (*p* < 0.05). (Table 3).

**TABLE 3:** Clinical efficacy evaluation of AECOPD patients in the PH group.

	Day 0	Day 7	*t/Z*	*P*	Mean differernce 95%CI
Symptoms and sign scores	12.76 ± 3.85	7.54 ± 3.03	−8.846	<0.001^a^	−5.220(-6.412, −4.027)
mMRC	3(1.5, 3)	2(0, 3)	−3.788	<0.001^b^	−0.659(-0.946, −0.371)
CAT	18 (12, 22)	11(7, 17)	−5.248	<0.001^b^	−6.122(-7.727, −4.517)
WBC(^10^9^L)	8.38 ± 2.63	7.50 ± 3.25	−1.956	0.060^a^	−1.213(-2.481, 0.055)
NEU%	72.72 ± 11.46	66.47 ± 10.61	−3.474	0.002^a^	−7.857(-12.482, −3.232)
CRP(mg/L)	11.05(1.64, 59.98)	5.37(2.02, 9.13)	−3.017	0.003^b^	−33.133(-54.283, −13.984)

^a^ Paired *t*-test.

^b^ Wilcoxon signed-rank test. mMRC, modified British Medical Research Council; CAT, COPD assessment test; WBC, white blood cell; NEU%, percentage of neutrophil count; CRP, C-reactive protein.

In the PD group, the clinical symptoms and sign, mMRC, and CAT scores of the patients after treatment were lower than before treatment (*p* < 0.05). The NEU% and CRP levels after treatment were significantly lower than those before treatment (*p* < 0.05). (Table 4).

**TABLE 4:** Clinical efficacy evaluation of AECOPD patients in the PD group.

	Day 0	Day 7	*t/Z*	*P*	Mean differernce 95%CI
Symptoms and sign scores	12.64 ± 4.30	7.93 ± 3.01	−8.552	<0.001^a^	−4.714(-5.845, −3.583)
mMRC	3(2, 3)	2(2, 3)	−3.500	<0.001^b^	−0.500(-0.724,-0.276)
CAT	17.5(13,24)	11.5(7, 17.75)	−4.162	<0.001^b^	−6.143(-7.817, −4.468)
WBC(^10^9^L)	6.56 ± 1.86	6.45 ± 2.30	−0.187	0.854^a^	−0.114(-1.380, 1.153)
NEU%	66.59 ± 11.28	61.94 ± 12.70	−2.180	0.041^a^	−5.890(-11.511, −0.270)
CRP(mg/L)	5.39(2.12, 30.52)	4.08(1.41, 14.79)	−1.979	0.048^b^	−27.40(-48.97, −5.82)

^a^ Paired *t*-test.

^b^ Wilcoxon signed-rank test. mMRC, modified British Medical Research Council; CAT, COPD assessment test; WBC, white blood cell; NEU%, percentage of neutrophil count; CRP, C-reactive protein.

### 3.3 Screening of differential metabolites

**FIGURE 1:**
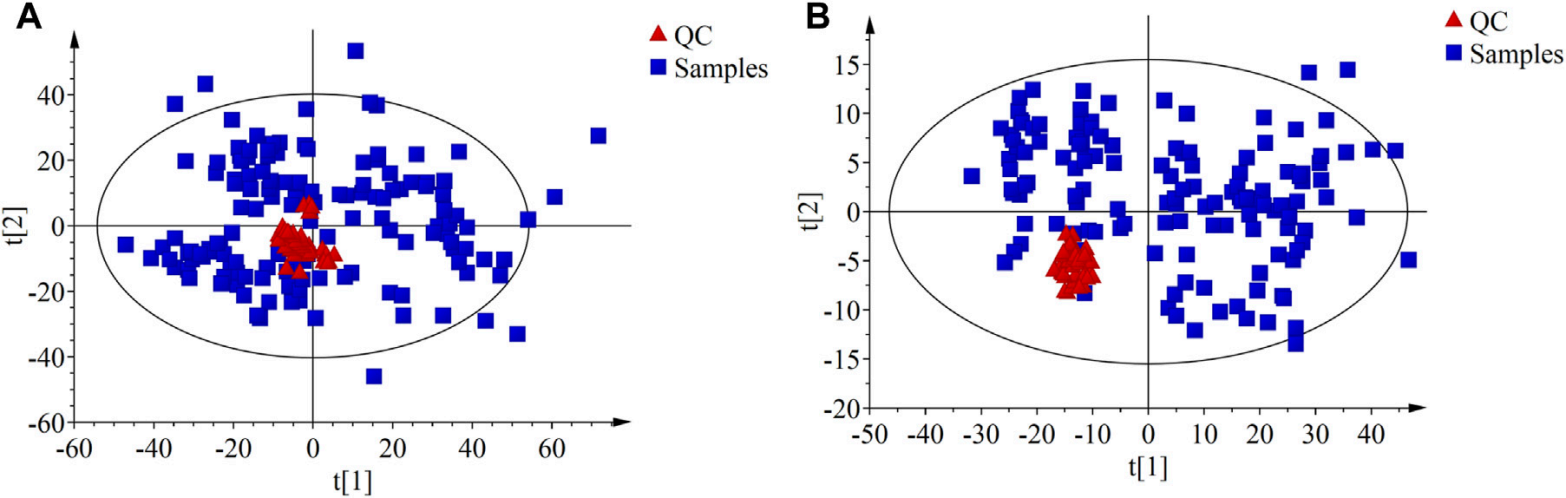
The principal component analysis (PCA) score plots compare the QC samples with all the study samples. **(A)** PCA score plot in ESI + mode; **(B)** PCA score plot in ESI- mode.

PCA was performed on the QC samples to evaluate data quality. The results showed that QC samples clustered well in both ESI+ and ESI- modes (as shown in Figure 1), indicating that the instrument system was stable and the analysis results had good reproducibility.

A total of 3,095 peaks in ESI + mode and 1,168 peaks in ESI- mode were extracted using Compound Discover 2.1. After removing compounds with a RSD larger than 30%, a total of 2,438 compounds in ESI + mode and 854 compounds in ESI- mode were obtained for statistical analysis. After the Wilcoxon signed-rank test, we screened 401 and 127 differential compounds for ESI+ and ESI- modes before and after treatment in the PH group, 366 and 162 differential compounds for ESI+ and ESI- modes, respectively, before and after treatment in the PD group (*p <* 0.05).

**FIGURE 2:**
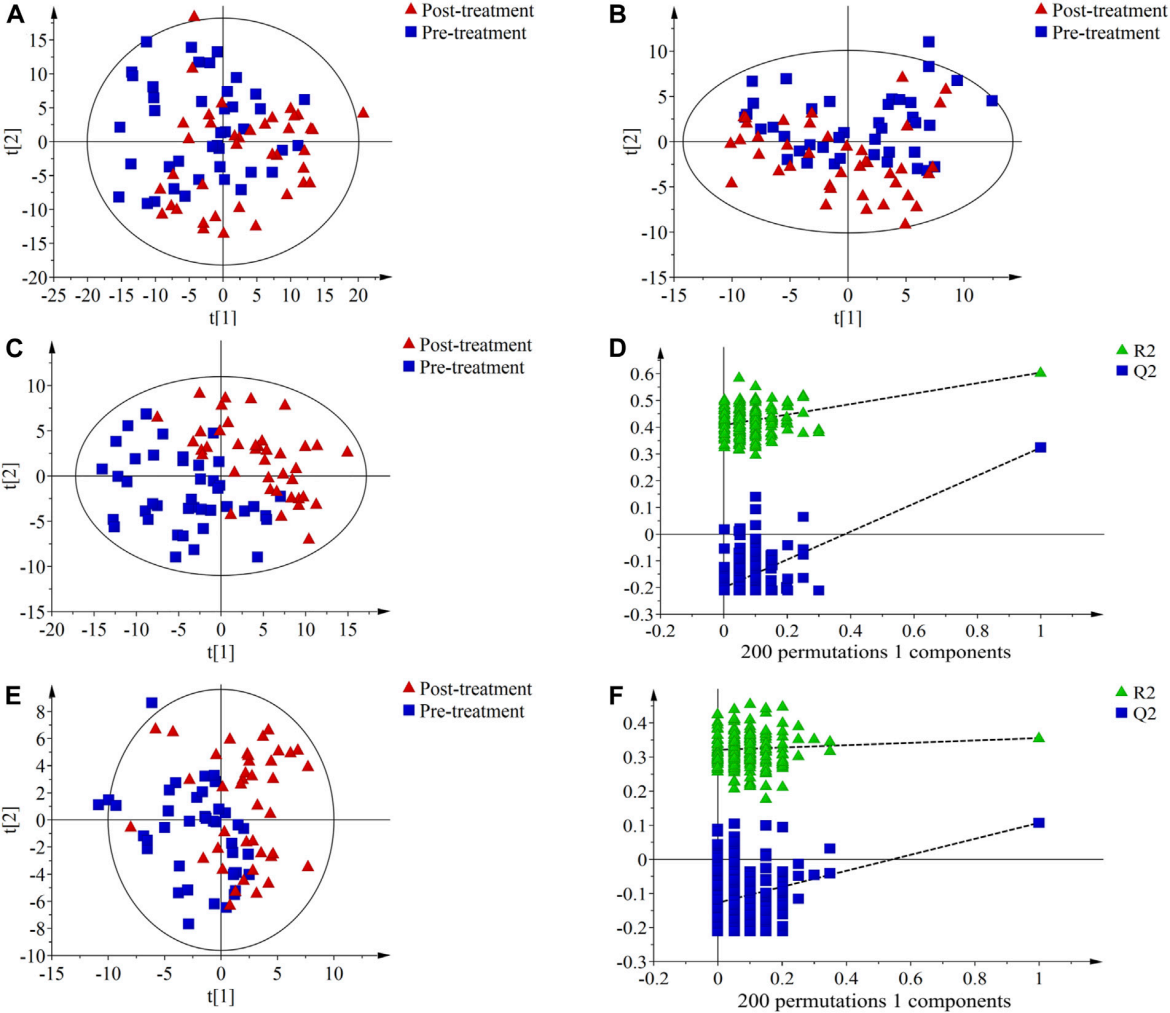
Principal component analysis (PCA) score plots, partial least squares discriminant analysis (PLS-DA) score plots and permutation test of serum metabolic profiling in pre-treatment (blue squares) and post-treatment (red triangles) of the PH group. **(A)** PCA score plot in ESI + mode; **(B)** PCA score plot in ESI- mode; **(C)** PLS-DA score plot in ESI + mode. The model parameters were R^2^X = 0.221, R^2^Y = 0.604, Q^2^ = 0.323; **(D)** Permutation test of PLS-DA score plot ESI + mode. The parameters were *R* ^2^ = 0.408, Q^2^ = −0.200; **(E)** PLS-DA score plot in ESI- mode. The model parameters were R^2^X = 0.353, R^2^Y = 0.355, Q^2^ = 0.107; **(F)** Permutation test of PLS-DA score plot ESI- mode. The parameters were *R* ^2^ = 0.321, Q^2^ = −0.127.

**FIGURE 3:**
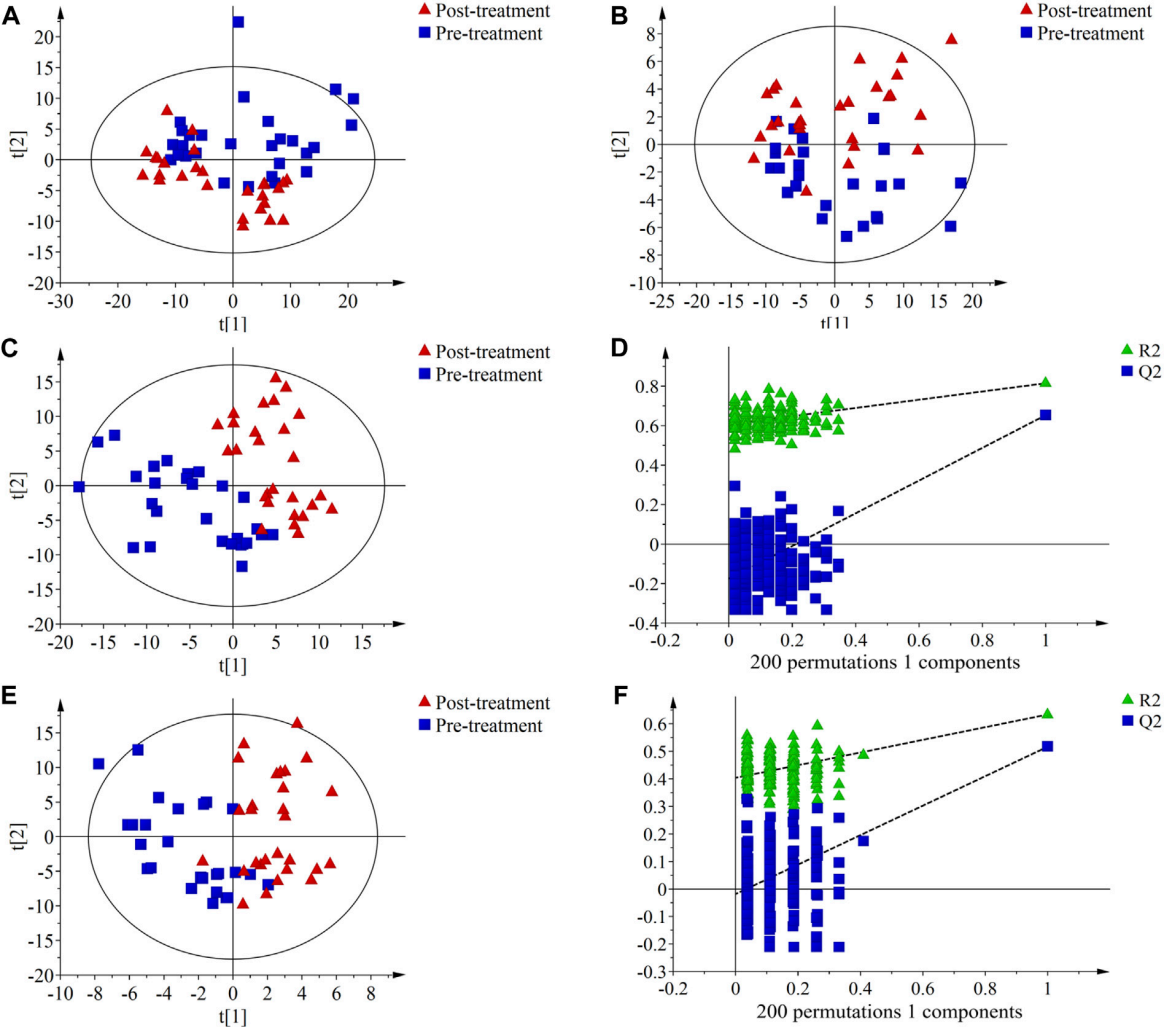
Principal component analysis (PCA) score plots, partial least squares discriminant analysis (PLS-DA) score plots and permutation test of serum metabolic profiling in pre-treatment (blue squares) and post-treatment (red triangles) of the PD group. **(A)** PCA score plot in ESI + mode; **(B)** PCA score plot in ESI- mode; **(C)** PLS-DA score plot in ESI + mode. The model parameters were R^2^X = 0.393, R^2^Y = 0.815, Q^2^ = 0.653; **(D)** Permutation test of PLS-DA score plot ESI + mode. The parameters were *R* ^2^ = 0.606, Q^2^ = −0.175; **(E)** PLS-DA score plot in ESI- mode. The model parameters were R^2^X = 0.456, R^2^Y = 0.634, Q^2^ = 0.517; **(F)** Permutation test plots of PLS-DA score plot ESI- mode. The parameters were *R* ^2^ = 0.404, Q^2^ = −0.019.

The results of PCA showed a certain degree of separation in serum samples before and after treatment in the PH (Figures 2A, B) and PD groups (Figures 3A, B), although the difference was not significant. PLS-DA was used to screen for differential metabolic compounds before and after treatment. As shown in Figures 2C, E and Figures 3C, E, the serum samples before and after treatment in each group were clearly separated. The PLS-DA models were further validated using permutation tests, and the results showed that the Q2 intercepts of the permutation tests were all less than zero, indicating that the PLS-DA models were valid and reliable without overfitting (Figures 2D, F; Figures 3D, F). According to the standard of VIP ≥1.00, 131 differential compounds (ESI+) and 37 differential compounds (ESI-) were screened out in the PH group, while 95 differential compounds (ESI+) and 36 differential compounds (ESI-) in the PD group before and after treatment respectively.

**FIGURE 4:**
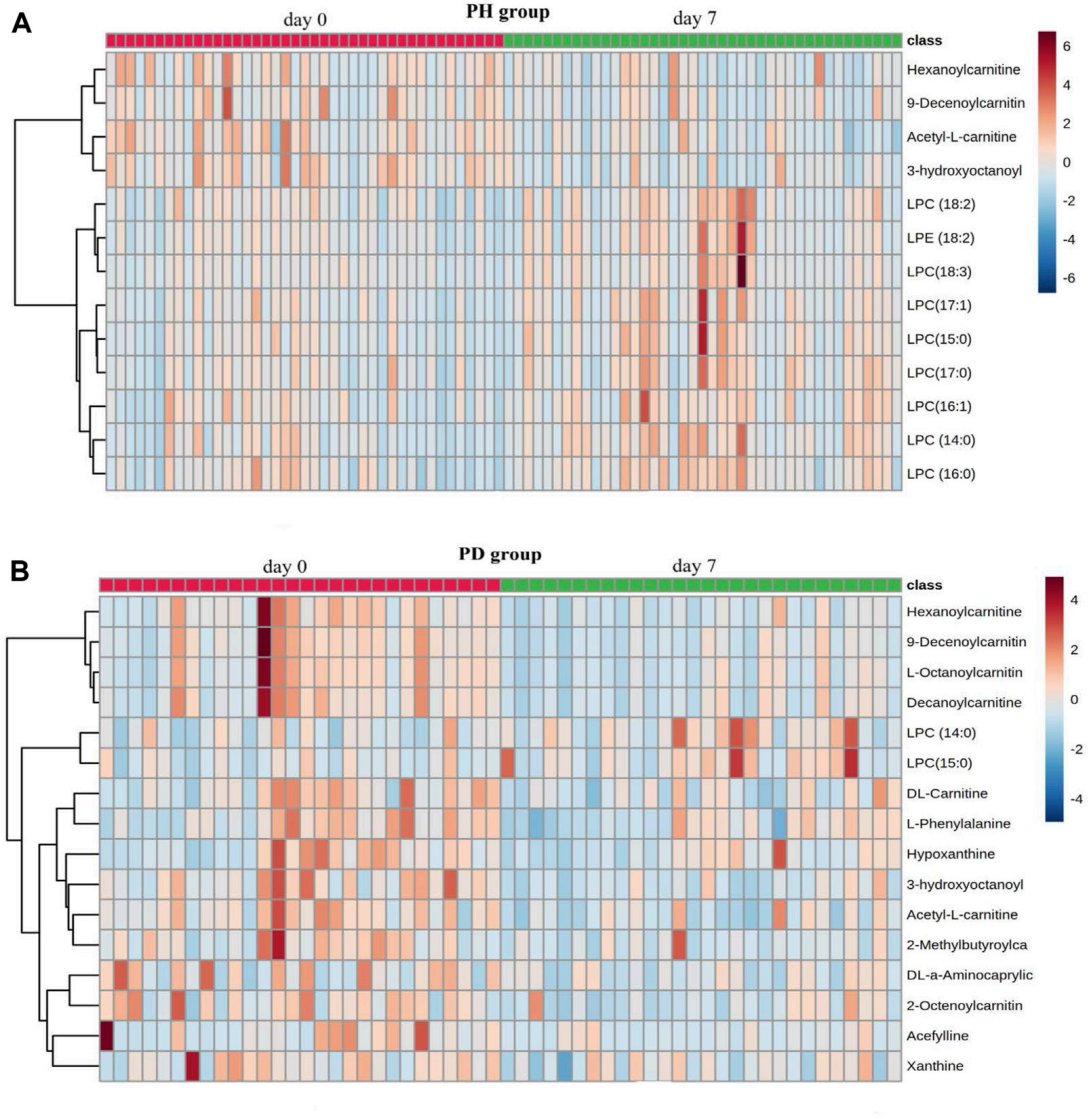
Heatmap of differential metabolites. **(A)** PH group; **(B)** PD group. The heatmap was created using the balanced log2-transformed and normalized data with 41 cases in the PH group and 28 cases in the PD group before and after treatment. Each column represents one sample. Columns with a red line on top represent samples before treatment, and the columns with a green line on top represent samples after treatment. In the subsequent rows, red hues represent increased concentrations, and blue hues decreased concentrations. Color intensity increases proportionally to the magnitude of the change.

By searching and comparing through various databases, a total of 13 different metabolites in the PH group (Table 5) and 16 different metabolites in the PD group (Table 6) before and after treatment were identified. The results of metabolites enrichment showed that in the PH group, the levels of lysophosphatidylcholine (LPC) (14:0), LPC (15:0), LPC (16:0), LPC (17:1), lysophosphatidylethanolamine (LPE) (18:2), LPC (18:3), LPC (18:2), LPC (16:1), and LPC (17:0) after treatment were upregulated compared to before treatment, while the levels of acetyl-L-carnitine, 3-hydroxyoctanoyl carnitine, hexanoylcarnitine, and 9-decenoylcarnitine after treatment were downregulated than before treatment. In the PD group, among the 16 differential metabolites before and after treatment, the levels of LPC (14:0) and LPC (15:0) were lower after treatment, whereas the levels of the other 14 differential metabolites increased after treatment (Figure 4).

**TABLE 5:** Differential metabolites in the PH group before and after treatment.

Metabolites	Molecular weight	RT (min)	VIP	*P* ^a^	Trend
Acetyl-L-carnitine	203.11626	0.937	1.58	<0.001	↓
3-hydroxyoctanoyl carnitine	303.20513	7.689	1.45	<0.001	↓
LPC(14:0)	467.30216	15.811	1.42	<0.001	↑
LPC(15:0)	481.31779	16.894	1.35	0.002	↑
LPC(16:0)	495.33354	17.579	1.32	0.003	↑
LPC(17:1)	507.33354	17.434	1.31	0.001	↑
LPE (18:2)	477.28644	16.661	1.25	0.004	↑
LPC(18:3)	517.31792	15.983	1.23	0.002	↑
LPC(18:2)	519.33364	16.739	1.20	0.032	↑
LPC(16:1)	493.31783	16.524	1.19	0.001	↑
LPC(17:0)	509.34917	19.186	1.18	0.009	↑
Hexanoylcarnitine	259.17884	7.304	1.13	0.002	↓
9-Decenoylcarnitine	313.22583	11.954	1.05	0.007	↓

^a^ Wilcoxon signed-rank test. RT, retention time; VIP, variable importance projection; LPC, lysophosphatidylcholine; LPE, lysophosphatidylethanolamine.

**TABLE 6:** Differential metabolites in the PD group before and after treatment.

Name	Molecular weight	RT (min)	VIP	*P* ^a^	Trend
Xanthine	152.03206	0.866	1.59	0.004	↓
Hexanoylcarnitine	259.17884	7.304	1.54	<0.001	↓
3-hydroxyoctanoyl carnitine	303.20513	7.689	1.50	<0.001	↓
9-Decenoylcarnitine	313.22583	11.954	1.45	<0.001	↓
Acetyl-L-carnitine	203.11626	0.937	1.44	0.002	↓
Decanoylcarnitine	315.24157	13.043	1.42	<0.001	↓
L-Octanoylcarnitine	287.21028	10.426	1.42	<0.001	↓
Acefylline	238.06975	1.652	1.34	0.010	↓
2-Octenoylcarnitine	285.19453	8.923	1.17	<0.001	↓
LPC(15:0)	481.31779	16.894	1.16	0.001	↑
Hypoxanthine	136.03888	0.918	1.14	0.024	↓
L-Phenylalanine	165.07940	2.060	1.13	<0.001	↓
2-Methylbutyroylcarnitine	245.16327	5.119	1.13	0.001	↓
LPC(14:0)	467.30216	15.811	1.11	0.003	↑
DL-α-Aminocaprylic acid	159.12634	5.670	1.08	0.024	↓
DL-Carnitine	161.10554	0.790	1.02	0.002	↓

^a^ Wilcoxon signed-rank test; RT, retention time; VIP, variable importance projection; LPC, lysophosphatidylcholine.

### 3.4 Analysis of metabolic pathways

**FIGURE 5:**
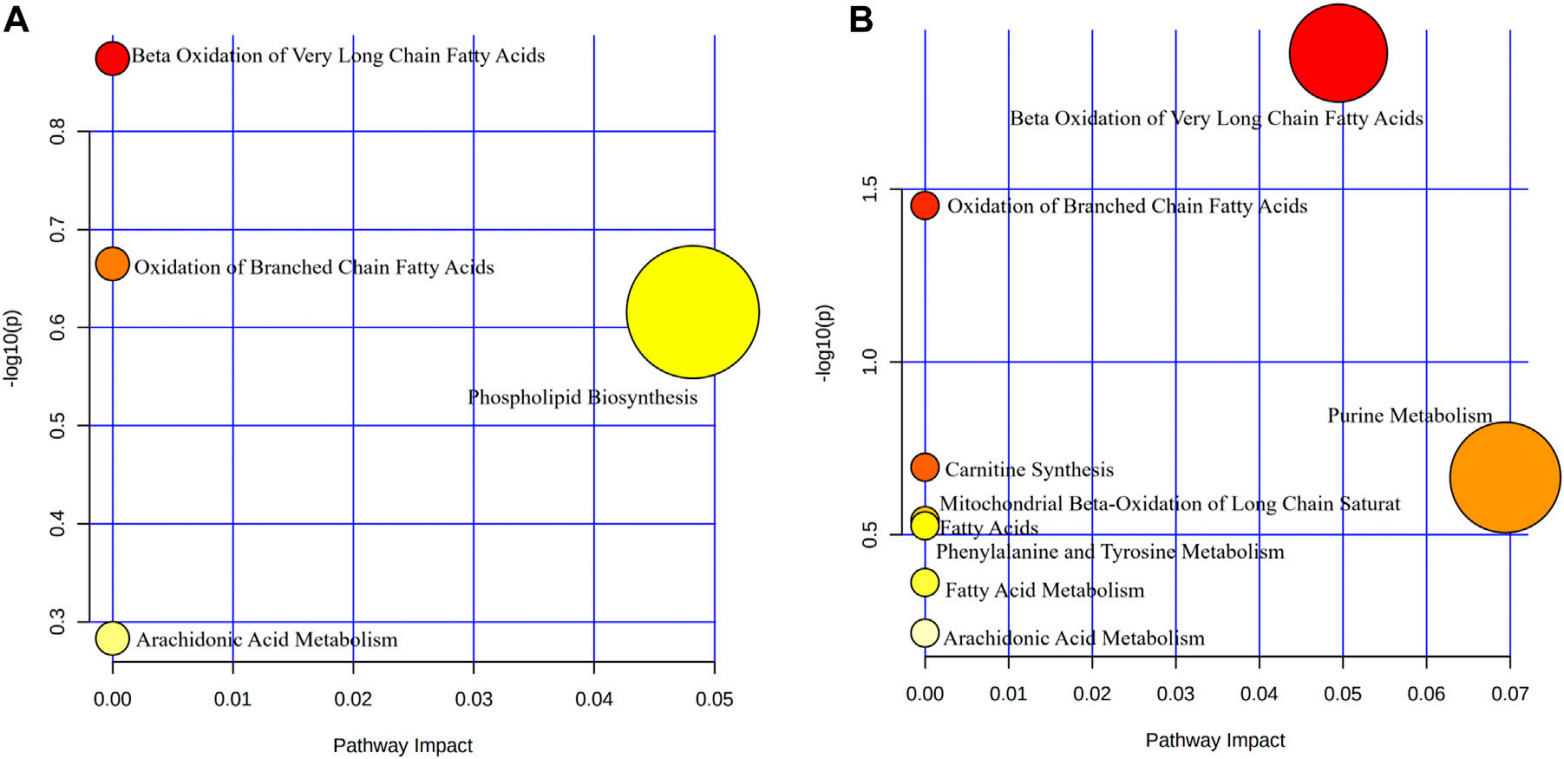
Bubble map of metabolic pathway enrichment. Bubble size indicates the number of differential metabolites involved in this pathway. The bubble color represents the *p*-value of this metabolic pathway. **(A)** PH group; **(B)** PD group.

Pathway enrichment analysis of differential metabolite showed that the metabolism involved in treatment in the PH group mainly included β-oxidation of very long-chain fatty acids, oxidation of branched-chain fatty acids, phospholipid biosynthesis, and arachidonic acid metabolism (Figure 5A). The metabolic pathways involved in treatment in the PD group were β-oxidation of very long-chain fatty acids, oxidation of branched-chain fatty acids, carnitine synthesis, purine metabolism, mitochondrial β-oxidation of long chain saturated fatty acids, phenylalanine, and tyrosine metabolism, fatty acid metabolism, arachidonic acid metabolism (Figure 5B).

### 3.5 Correlation of differential metabolites and indicators of efficacy evaluation

In the PH group, LPC (15:0) (r = −0.403, *p* < 0.01), LPC (16:0) (r = −0.381, *p* < 0.05), LPC (17:1) (r = −0.470 *p <* 0.01), LPC (18:3) (r = −0.366, *p* < 0.05), LPC (18:2) (r = −0.311, *p* < 0.05), and LPC (17:0) (r = −0.405, *p* < 0.01) significantly negatively correlated with clinical symptoms and sign scores. Hexanoylcarnitine (r = 0.477, *p* < 0.01) and 9-decenoylcarnitine (r = 0.427, *p* < 0.01) levels negatively correlated with mMRC. LPC (16:0) (r = −0.494, *p* < 0.01), LPC (17:1) (r = −0.470, *p* < 0.01), LPC (16:1) (r = −0.495, *p <* 0.01), and LPC (17:0) (r = −0.484, *p* < 0.01) negatively correlated with WBC. LPC (16:0) (r = −0.457, *p* < 0.05), LPC (17:1) (r = −0.494, *p* < 0.01), LPC (16:1) (r = −0.461, *p* < 0.05), and LPC (17:0) (r = −0.426, *p* < 0.05) negatively correlated with NEU%. LPC (16:0) (r = −0.372, *p* < 0.05) negatively correlated with CRP levels. There was no significant correlation between the other differential metabolites and efficacy evaluation indicators in the phlegm heat group (Table 7).

**TABLE 7:** Correlation of differential metabolites and efficacy evaluation index in the PH group.

Metabolites	Symptoms and sign scores	CAT	mMRC	WBC	NEU%	CRP
Acetyl-L-carnitine	0.049	0.008	0.225	0.107	−0.011	−0.155
3-hydroxyoctanoyl carnitine	0.055	0.115	0.308	−0.029	−0.089	−0.069
LPC(14:0)	−0.302	−0.273	−0.139	−0.152	−0.201	−0.128
LPC(15:0)	−0.403^**^	−0.268	−0.148	−0.315	−0.285	−0.255
LPC(16:0)	−0.381^*^	−0.274	−0.004	−0.494^**^	−0.457^*^	−0.372^*^
LPC(17:1)	−0.470^**^	−0.281	−0.025	−0.470^**^	−0.494^**^	−0.280
LPE(18:2)	−0.286	−0.274	−0.177	−0.160	−0.257	−0.075
LPC(18:3)	−0.366^*^	−0.207	−0.108	−0.283	−0.315	−0.168
LPC(18:2)	−0.311^*^	−0.199	−0.009	−0.321	−0.269	−0.118
LPC(16:1)	−0.303	−0.241	−0.055	−0.495^**^	−0.461^*^	−0.187
LPC(17:0)	−0.405^**^	−0.211	0.071	−0.484^**^	−0.426^*^	−0.305
Hexanoylcarnitine	0.216	0.158	0.477^**^	0.078	0.012	−0.038
9-Decenoylcarnitine	0.165	0.159	0.427^**^	−0.013	−0.007	−0.023

Spearman correlation analysis:^**^ *p* < 0.01. *p < 0.05.

In the PD group, correlation analysis between differential metabolites and indicators of efficacy evaluation showed that LPC (15:0) (r = −0.393, *p* < 0.05) and LPC (14:0) (r = −0.563, *p* < 0.01) negatively correlated with clinical symptoms and sign scores. 2-Methylbutyroylcarnitine (r = 0.480, *p* < 0.01) and DL-carnitine (r = 0.459, *p* < 0.05) positively correlated with clinical symptoms and sign scores. Acefylline (r = 0.382, *p* < 0.05) and 2-methylbutyroylcarnitine (r = 0.387, *p* < 0.05) positively correlated with mMRC. LPC (15:0) negatively correlated with CAT scores (r = 0.388, *p* < 0.05). LPC (15:0) (r = −0.444, *p* < 0.05) negatively correlated with CRP levels. L-phenylalanine (r = 0.463, *p* < 0.05) positively correlated with CRP levels. The remaining differential metabolites in the PD group were not significantly correlated with the efficacy indicators (Table 8).

**TABLE 8:** Correlation of differential metabolites and efficacy evaluation index in the PD group.

Metabolites	Symptoms and sign scores	CAT	mMRC	WBC	NEU%	CRP
Xanthine	−0.110	−0.247	0.249	−0.146	0.138	−0.237
Hexanoylcarnitine	0.326	0.002	−0.159	−0.135	0.033	−0.026
3-hydroxyoctanoyl carnitine	0.300	−0.096	−0.314	−0.186	−0.146	−0.054
9-Decenoylcarnitine	0.286	0.039	−0.201	−0.108	0.024	0.169
Acetyl-L-carnitine	0.351	0.122	−0.082	0.078	0.124	0.032
Decanoylcarnitine	0.207	−0.118	−0.327	−0.131	0.092	0.142
L-Octanoylcarnitine	0.236	−0.095	−0.277	−0.191	−0.007	0.022
Acefylline	0.254	0.382^*^	−00.170	0.340	0.308	0.180
2-Octenoylcarnitine	0.089	0.106	−0.127	−0.400	−0.412	−0.343
LPC(15:0)	−0.393^*^	−0.292	0.388^*^	−0.226	−0.369	−0.444^*^
Hypoxanthine	0.177	0.096	−0.045	−0.152	−0.208	−0.157
L-Phenylalanine	0.212	0.131	−0.069	−0.063	0.261	0.463^*^
2-Methylbutyroylcarnitine	0.480^**^	0.387^*^	−0.064	−0.046	−0.155	0.189
LPC(14:0)	−0.563^**^	−0.271	0.299	−0.221	−0.315	−0.303
DL-α-Aminocaprylic acid	0.021	0.023	0.121	−0.084	−0.292	−0.393
DL-Carnitine	0.459^*^	0.138	−0.140	−0.005	0.197	0.054

Spearman correlation analysis: **p < 0.01. *p < 0.05.

## 4 Discussion

With the wide application of metabolomics technology to improve the objectivity and accuracy of TCM syndrome diagnosis and efficacy evaluation, research on biomarkers based on metabolomics has become a popular topic. Zhang et al. found that the expression of 15 substances including lactic acid, succinic acid, lipoic acid, neohesperid, D-erythro-sphingosine, glucose, methyl linoleate, asparagine, and urea was significantly upregulated, while threonine and taurine were significantly downregulated in patients with AECOPD phlegm-turbid syndrome (Zhang et al., 2020). Another study found that there are 50 kinds of small-molecule differential metabolites in the serum of patients with the syndrome of phlegm-heat congesting lung and syndrome of non-phlegm-heat congesting lung of AECOPD, involving metabolic disorders such as sphingolipid, glycerophospholipid, and linoleic acid metabolisms (Sun, 2019). Although several studies have shown that metabolites in patients with different TCM syndromes of AECOPD are different from those in the stable stage or in healthy people, there are still few reports on the biomarkers related to the efficacy evaluation of TCM syndromes. Therefore, on the basis of evaluating the clinical efficacy of syndrome differentiation and treatment of TCM combined with routine treatment of Western medicine, this study conducted a metabolomic analysis of the serum of AECOPD patients with the syndromes of PH and PD through metabolomics and initially explored the biomarkers related to the evaluation of TCM syndrome efficacy of AECOPD, providing an objective basis for the evaluation of syndrome efficacy.

In this study, 23 differential metabolites were screened and identified. Among them, LPC (14:0), LPC (15:0), 3-hydroxyoctanoyl carnitine, hexanoylcarnitine, acetyl-L-carnitine, and 9-decenoylcarnitine were common metabolites in both groups, and the trends of these metabolites was consistent before and after treatment in both groups. This may be due to changes in the metabolic profile caused by conventional Western medicine treatment. Additionally, acefylline is a metabolite of the bronchodilator doxofylline; therefore, it is not considered a biomarker of TCM syndrome efficacy.

In the PH group, the characteristic differential metabolites were LPCs, among them LPC (16:0) and LPC (17:0) are saturated acyl LPCs, and LPC (16:1), LPC (17:1), LPC (18:2), and LPC (18:3) are unsaturated acyl LPCs. Combined with the correlation analysis of clinical efficacy evaluation indicators, we speculated that LPC (16:0), LPC (17:0), LPC (16:1), LPC (17:1), LPC (18:2), and LPC (18:3) may be potential biomarkers of AECOPD with syndrome of PH. These LPCs are involved in glycerophospholipid metabolism and arachidonic acid metabolism, and the serum levels post-treatment of these LPCs were higher than the pre-treatment levels. This result is consistent with a previous study, that showed that the concentrations of serum LPCs, such as LPC (14:0), LPC (16:0), LPC (16:1), LPC (18:0), LPC (18:2), and LPC (18:3) in patients with AECOPD gradually increases after 10–14 days of conventional treatment (Gai et al., 2021). Studies have shown that LPC concentrations vary in different diseases. For example, the concentration of LPC increases in inflammatory diseases (Liu et al., 2020) or adrenoleukodystrophy (Huffnagel et al., 2017), but decreases in cancer (Kim et al., 2014) or infectious diseases (Ahn et al., 2017). LPCs not only exert a pro-inflammatory effect by activating a variety of signaling pathways involved in oxidative stress and inflammation, but also play an anti-inflammatory and anti-infective effects by enhancing natural immunity and adaptive immune responses (Piccirillo et al., 2019; Liu et al., 2020). The pro-inflammatory activity of saturated acyl LPC, such as LPC (16:0), can be antagonized by polyunsaturated acyl LPC, such as LPC (16:1) (Hung et al., 2012; Liu et al., 2020). The pathological change of COPD is chronic airway inflammation and AECOPD is typically associated with increased local or systemic inflammation caused by respiratory infections and air pollution (Expert group on Acute Exacerbations of Chronic Obstructive Pulmonary Disease, 2017; Christenson et al., 2022; GOLD, 2023). Abnormalities in arachidonic acid metabolism, glycerophospholipid metabolism, glycosylphosphatidylinositol-anchored biosynthesis, and linoleic acid metabolism may be related to chronic airway inflammation, vascular endothelial contraction, oxidative stress, and energy depletion in patients with AECOPD. Among the various AECOPD syndromes, the inflammatory reaction in the syndrome of PH is the most intense. Sphingomyelin metabolism and the glycerol phospholipid metabolic pathway may constitute the metabolic pathways of AECOPD PH syndrome disturbances (Sun, 2019). In this study, TCM syndrome differentiation and treatment achieved a therapeutic effect by correcting the disorders of glycerol phospholipid and arachidonic acid metabolism caused by AECOPD, which is consistent with the results of previous studies.

In the PD group, most of the differential metabolites were medium-chain acyl carnitines, such as hexanoylcarnitine, 3-hydroxyoctanoyl carnitine, acetyl-L-carnitine, decanoylcarnitine, 2-octenoylcarnitine, L-octanoylcarnitine, 2-methylbutyroyl carnitine, and 9-decenoylcarnitine, which play key roles in lipid metabolism and beta-oxidation. Correlation analysis showed that DL-carnitine was significantly and positively correlated with clinical symptoms and sign scores and 2-methylbutyroyl carnitine was positively correlated with clinical symptoms and sign scores, and CAT scores. Carnitine, a non-essential amino acid and a quaternary ammonium compound, is concentrated in skeletal and cardiac muscles, as well as in other tissues that metabolize fatty acids as an energy source. The main role of carnitine is to assist in the transportation of fatty acids to the mitochondria for β-oxidation and energy supply. During this process, the intermediate metabolite fatty acyl carnitine, which play an important role in the transport and oxidation of fatty acids, is produced. Acetyl-L-carnitine, hexanoylcarnitine, and 3-hydroxyoctanoyl carnitine are essential for fatty acid metabolism (Capitanio et al., 2020). Carnitine has two isomers (D-carnitine and L-carnitine), both of which have biological activity; however, only L-carnitine is naturally present in animals, whereas D-carnitine is toxic, because it inhibits the activity of the L-forms. Most carnitine in the body comes from dietary sources such as red meat and dairy products. Plasma carnitine concentrations correlate with the nutritional intake of carnitine in healthy adults (Lennon et al., 1986; Steiber et al., 2004). Lower levels of L-carnitine indicate more severe the heart failure, and a higher degree of inflammation, and severe arrhythmia can quickly consume stored carnitine (Vescovo et al., 2005; Dambrova et al., 2021). In addition to β oxidation, carnitine can also act as a free radical scavenger to reduce oxidative stress-induced apoptosis. Reinke, S. N. et al. through a metabolomic analysis of the urine and sputum of patients with asthma, found that carnitines showed the greatest variation associated with asthma severity (Reinke et al., 2022). In patients with COPD, the carnitine level is insufficient, and the degree of deficiency is related to the severity of COPD (Conlon et al., 2016). In the AECOPD, due to hypoxia, the heart rate and oxygen consumption of the myocardium and peripheral respiratory muscles increase. This causes the carnitine stored in the body to be consumed rapidly, ultimately leading to a decrease in carnitine content. Our study showed that after treatment of 7 days, the carnitine content was lower than before treatment. A study showed that changes in carnitine levels in patients with asthma are not affected by glucocorticoid therapy (Reinke et al., 2022). Therefore, the reduction of carnitine levels in patients with AECOPD after treatment in our study may be related to the effect of Chinese medicine components, which may be further studied and verified as the target of Chinese medicine treatment in the future.

In the PD group, we detected another potential biomarker, L-phenylalanine, an alpha-amino acid, which is highly concentrated in many high-protein foods such as meat; L-phenylalanine is one of the 20 amino acids required for protein synthesis, involved in phenylalanine and tyrosine metabolism, and is present in all organisms. In this study, the post-treatment serum phenylalanine level was lower than the pre-treatment level, which is inconsistent with the results of a previous study in which the level of plasma phenylalanine in patients with AECOPD was lower than that in the stable stage (Inoue and Ikeda, 2019). The reason for this inconsistency may be the small sample size or grouping of TCM syndromes. In addition, we analyzed the correlation between L-phenylalanine and curative effects, and inflammatory indicators. The results showed that L-phenylalanine was significantly and positively correlated with CRP levels. Based on the above findings, L-carnitine, 2-methylbutyroyl carnitine, and L-phenylalanine may be potential biomarkers related to the efficacy of AECOPD with syndrome of PD.

In this study, there are still some limitations, such as not elucidating the detailed mechanism of action of these biomarkers. In future studies, network pharmacology and multi-omics combination methods will be used to further validate these potential markers and their mechanisms of action. This can not only provide an objective index for the evaluation of the curative effect of AECOPD syndrome differentiation of TCM, but also provide guidance for improving the composition of TCM prescriptions and improving clinical efficacy.

## 5 Conclusion

The findings of our study showed that the differential metabolites of the PH and PD groups were significantly different, indicating that the effects of syndrome differentiation treatment on the changes in the metabolism of patients with different TCM syndromes are different. The treatment of the syndrome of phlegm-heat congesting lung by Qingre Huatan granules mainly involves glycerophospholipid metabolism, and LPC (16:0), LPC (16:1), LPC (17:0), LPC (17:1), LPC (18:2), and LPC (18:3) may be potential biomarkers related to the efficacy of syndrome of PH. The treatment of the syndrome of phlegm-damp amassing lung by Zaoshi Huatan granules mainly involves fatty acid oxidation and carnitine biosynthesis, and L-carnitine, 2-methylbutyroyl carnitine, and L-phenylalanine may be potential biomarkers related to the efficacy of syndrome of PD.

## Data Availability

The raw data supporting the conclusion of this article will be made available by the authors, without undue reservation.
