# Supplementary material for: Exploration of biomarkers for efficacy evaluation of traditional Chinese medicine syndromes of acute exacerbation of chronic obstructive pulmonary disease based on metabolomics
Source: Front Pharmacol. 2024 Jan 26;15:1302950. doi: 10.3389/fphar.2024.1302950 (PMC10853405; doi:10.3389/fphar.2024.1302950)
Supplement: Supplementary file 1 [file Table1.DOCX]

Supplementary Material

Supplementary Table 1. Treatment of AECOPD patients without respiratory failure

| Patient education | Check the inhalation technology and consider the application of fog storage tank device |
| --- | --- |
| Bronchodilator | Short-acting β_2_ receptor agonists and / or quantitative inhalation of ipratropium bromide in fog storage tanks or humidifiers, or consider adding long-acting bronchodilators |
| Glucocorticoid  (actual application dose may vary) | Prednisone 30~40mg/d, oral administration for 9~14d;  Consider the use of inhaled corticosteroids (using different inhalation devices and techniques) |
| Antibiotics | It is recommended to use antibiotics and start antimicrobial therapy according to the changes of sputum characteristics of patients;  Antibiotics should be selected according to the drug resistance of local bacteria. |

Supplementary Table 2. Treatment of hospitalization in general ward of patients with severe AECOPD (non-life-threatening acute respiratory failure)

| Oxygen therapy and serial measurement of arterial blood gas, venous blood gas and pulse oxygen saturation measurement | |
| --- | --- |
| Bronchodilator | Increase the dose and/or frequency of short-acting bronchodilators;  Combined use of short-acting beta agonists and anticholinergic drugs;  When the condition stabilizes, consider using long-acting bronchodilator;  Application of fog accumulator or pneumatic atomization device. |
| Glucocorticoid | It is recommended to take oral glucocorticoids, prednisone 30~40mg/d, 9~14d;  Consider quantitative inhalation or aerosol inhalation of corticosteroids; |
| Antibiotics | Consider the use of antibiotics when there are signs of bacterial infection |
| Mechanical ventilation | If there is acute respiratory failure or acute exacerbation of chronic respiratory failure, non-invasive ventilation is recommended. |
| Pay attention at all times | Monitor liquid balance and nutrition;  Consider subcutaneous injection of heparin or low molecular weight heparin to prevent thrombosis;  Identification and treatment of complications (heart failure, arrhythmia, pulmonary embolism, etc.) |


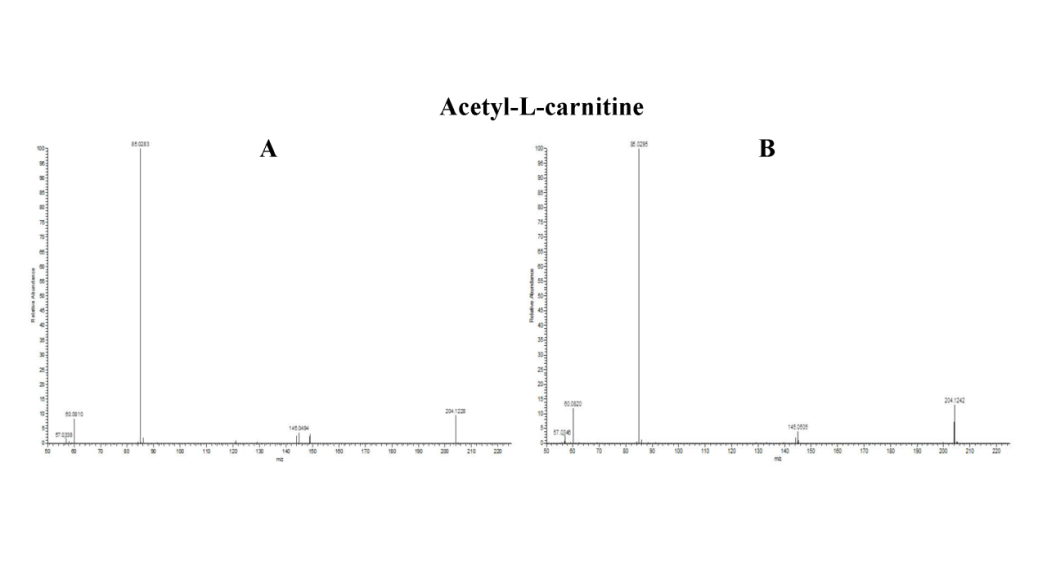


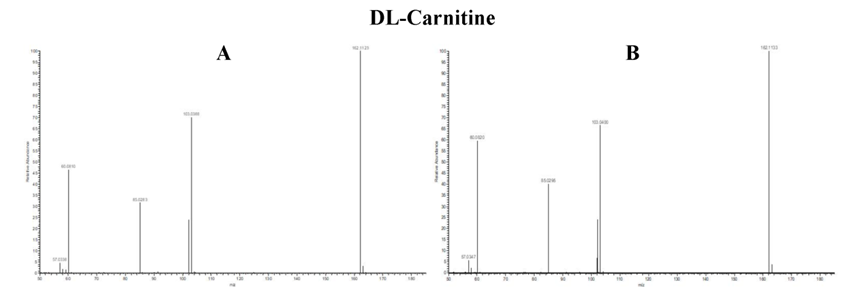


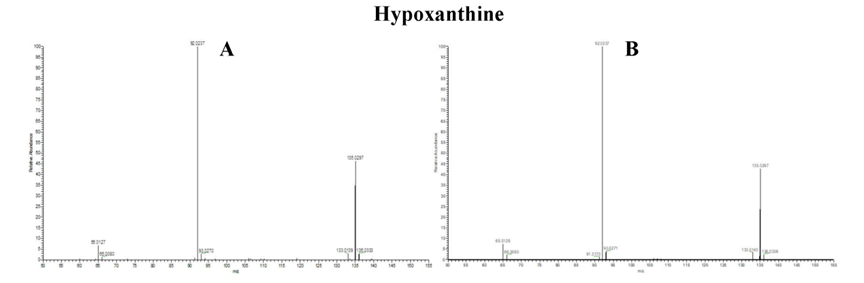


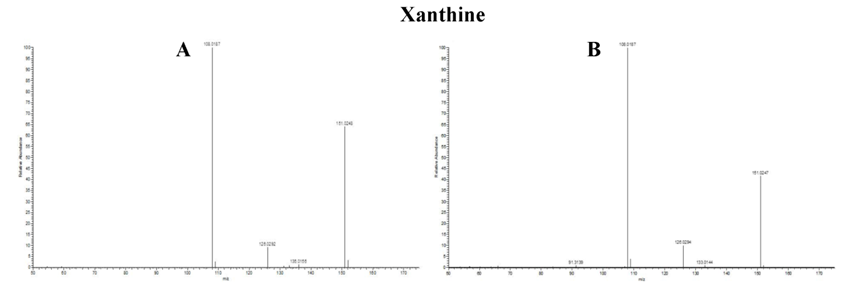


**Supplementary Figure 1.** The representative MS/MS spectra of four differential metabolites (Acetyl-L-carnitine, DL-Carnitine, Hypoxanthine, Xanthine). (A) The MS/MS spectra of standard sample spectra. (B) The MS/MS spectra of the serum sample
